# Enhanced Antitumor Response to Immune Checkpoint Blockade Exerted by Cisplatin-Induced Mutagenesis in a Murine Melanoma Model

**DOI:** 10.3389/fonc.2021.701968

**Published:** 2021-07-06

**Authors:** Falih M. Gorgun, Steven G. Widen, Douglas S. Tyler, Ella W. Englander

**Affiliations:** ^1^ Department of Neurosurgery, University of Texas Medical Branch, Galveston, TX, United States; ^2^ Department of Biochemistry and Molecular Biology, University of Texas Medical Branch, Galveston, TX, United States; ^3^ Department of Surgery, University of Texas Medical Branch, Galveston, TX, United States

**Keywords:** cisplatin, CD8 T lymphocyte, immune checkpoint blockade, lymph node, melanoma, mutagenesis, tumor regression

## Abstract

Sequencing data from different types of cancers including melanomas demonstrate that tumors with high mutational loads are more likely to respond to immune checkpoint blockade (ICB) therapies. We have previously shown that low-dose intratumoral injection of the chemotherapeutic DNA damaging drug cisplatin activates intrinsic mutagenic DNA damage tolerance pathway, and when combined with ICB regimen leads to tumor regression in the mouse YUMM1.7 melanoma model. We now report that tumors generated with an *in vitro* cisplatin-mutagenized YUMM1.7 clone (YUMM1.7-CM) regress in response to ICB, while an identical ICB regimen alone fails to suppress growth of tumors generated with the parental YUMM1.7 cells. Regressing YUMM1.7-CM tumors show greater infiltration of CD8 T lymphocytes, higher granzyme B expression, and higher tumoral cell death. Similarly, *ex-vivo*, immune cells isolated from YUMM1.7-CM tumors-draining lymph nodes (TDLNs) co-incubated with cultured YUMM1.7-CM cells, eliminate the tumor cells more efficiently than immune cells isolated from TDLNs of YUMM1.7 tumor-bearing mice. Collectively, our findings show that *in vitro* induced cisplatin mutations potentiate the antitumor immune response and ICB efficacy, akin to tumor regression achieved in the parental YUMM1.7 model by ICB administered in conjunction with intratumoral cisplatin injection. Hence, our data uphold the role of tumoral mutation burden in improving immune surveillance and response to ICB, suggesting a path for expanding the range of patients benefiting from ICB therapy.

## Introduction

Differences in tumoral mutational burden have emerged as predictors of clinical outcomes of immune checkpoint blockade (ICB) therapies across different cancers (1, 2). This notion has been informed by large data sets which demonstrated that mutations acquired during tumorigenesis can be translated into neoantigens that elicit antitumor immune responses, which subsequently are bolstered by ICB (3, 4). In view of these findings we reasoned that intratumoral delivery of low-dose DNA damaging agent calibrated to activate the tumoral mutagenic DNA damage tolerance pathway (5, 6), without causing extensive tumoral cell death, might be a productive route to increase tumoral neoantigen formation and reinvigorate the antitumor immune response (2, 7–10). Activation of the DNA damage tolerance pathway involves recruitment of error-prone translesion synthesis (TLS) DNA polymerases (6, 11). Using the YUMM1.7 melanoma mouse model (12), we have previously demonstrated that tumoral TLS polymerases are transiently elevated following intratumoral delivery of the DNA damaging chemotherapeutic drug, cisplatin (13) and that the anti-CTLA-4/anti-PD-1 ICB regimen (14, 15), given in conjunction with intratumoral cisplatin, leads to complete tumor regression in the mouse (13). These findings are consistent with the notion that low-dose chemotherapeutic treatments that increase tumoral mutation burden contribute to formation of immunogenic neoantigens and thereby provide a plausible path for enhancing the host’s antitumor immune response and improving immunotherapy outcomes. Notably, this targeted approach affords an important advantage over conventional chemotherapy, because tightly calibrated intratumoral delivery of a chemotherapeutic drug allows for significant dose reduction limiting debilitating generalized toxicity, while achieving durable desired antitumor effect *via* revitalized immune response.

To probe further the involvement of cisplatin-induced mutagenesis in achieving the desired outcomes of combination treatments, we asked in the current study whether this scenario could be recapitulated with the *in vitro* cisplatin-mutagenized YUMM1.7-CM clone-generated tumors. We found that the YUMM1.7-CM clone produced by *in vitro* exposure of YUMM1.7 cells to low concentration of cisplatin, acquired non-synonymous mutations, as well as phenotypic changes, including increased cell dimensions and reduced doubling time. Notably, compared to parental YUMM1.7 cells, the mutagenized YUMM1.7-CM cells were more efficiently eliminated when co-cultured *ex-vivo* with immune cells isolated from the YUMM1.7-CM tumors draining lymph nodes. We also detected higher levels of infiltrating CD8 T lymphocytes with higher granzyme B expression in the YUMM1.7-CM generated tumors and most importantly, suppression of tumor growth in response to anti-CTLA-4/anti-PD-1 checkpoint blockade regimen. Considered together, our findings show that the *in vitro* cisplatin-mutagenized YUMM1.7-CM melanoma clone-generated tumors respond to ICB regimen similarly to the parental YUMM1.7-generated tumors, subjected *in vivo* to intratumoral cisplatin injection, supporting a pivotal role for increases in tumoral mutational loads in augmentation of the host’s antitumor immune response.

## Methods

### Cell Culture and Cisplatin Exposure

YUMM1.7 mouse melanoma cells (12) were purchased from ATCC (ATCC CRL-3362) and cultured in DMEM/F12 (Invitrogen #11320033) with 10% FBS (ATCC #30-2020), 1% non-essential amino acid (Gibco #11440-076), and 1% penicillin/streptomycin. Cells were maintained at confluence below 85% and were routinely inspected for Mycoplasma (Lonza MycoAlert Kit #LT07-118) as we described (16). YUMM1.7 cells exposure to 0.2 µM cisplatin was initiated 24 h post seeding; cells were cultured in the presence of cisplatin for 5 weeks with routine refeeding by replacement of half medium volume twice/week and splitting at 1:2 as needed. Extended cisplatin exposure caused cell flattening with progressive slowing of proliferation that came to near halt by 5 weeks. At that time, cells were harvested, seeded in cisplatin-free medium at ~1 cell/well in 96-well plates and cultured for 12 days. Wells with single clones were identified, collected, and expanded. Selected clones were passaged, and doubling times determined. A single YUMM1.7-Cisplatin Mutagenized (YUMM1.7-CM) clone was chosen for subsequent studies.

### Whole Exome Sequencing and Mutation Mapping

Library construction and sequencing: Genomic DNA was isolated from YUMM1.7 and YUMM1.7-CM melanoma cells using the Easy-DNA™ kit (K1800-01, Invitrogen). DNA was fragmented to less than 500 base pairs with a Covaris S220 instrument. Approximately 50 ng was used to prepare sequencing libraries with the NEBNext Ultra II DNA library kit (NEB #E7103, New England BioLabs) following the manufacturer’s protocol. Exome capture was performed with the Twist Biosciences (San Francisco, CA, USA) Mouse Exome Panel kit (#102035) following the suggested protocol. Briefly, ~400 ng of each library was pooled, dried in a vacuum concentrator, and dissolved in hybridization mix including the biotinylated exome capture probes, denatured, and incubated at 70°C for 16 h. Paramagnetic streptavidin beads were used to capture the hybridized material, the beads were then washed and placed directly into a PCR reaction to amplify the libraries. Following purification, the libraries were quantified and sequenced on NextSeq 550 Illumina System with the High Output flow cell and a 40-base paired-end protocol. Reads were demultiplexed and converted to FASTQ format with Illumina’s bcl2fast2 software. YUMM1.7 DNA had 242 million read pairs and YUMM1.7-CE 184 million (data are available at NCBI Bioproject PRJNA734588). Variant analysis: The Broad Institute Best Practices protocols for variant discovery using the tumor-normal methods, with the mutated cell line taking the place of the tumor sample, were followed (17). Reads were aligned to the mouse mm10 reference genome with the Burrows-Wheeler Aligner (BWA) alignment program (18), version 0.7.17, with the BWA-backtrack algorithm commands. The resulting BAM format files were pre-processed for variant discovery with the MarkDuplicates and BaseRecalibrator functions of GATK, version 4.1.9.0, with default parameters. The processed BAM files were input into the Mutect2 software with YUMM1.7 as the normal sample. The output variant call format (VCF) file was filtered with the FilterMutectCalls function with parameters –min-allele-fraction 0.15 and –unique-alt-read-count 3. Variants found in exon coding regions were inspected for amino acid changes.

### *In Vivo* Mouse Experiments

All mouse handling procedures were approved by Institutional Animal Care and Use Committee of the University of Texas Medical Branch, Galveston, Texas. Female 8–10-week-old C57BL/6 mice were purchased from Envigo (USA) and acclimated for 2 weeks. YUMM1.7 or YUMM1.7-CM tumors were generated by subcutaneous inoculation of 5 × 10^4^ cells suspended in a 2:1 PBS/solubilized Matrigel Membrane Matrix (#354234 Corning) into upper left hindlimb as we described (13). Tumor dimensions were recorded 3×/week using vernier calipers and the formula: volume = ([length] × [width]^2^)/2 to calculate the volume (19). Mice were IP injected with anti-PD-1 (clone RMP1-14, BE0146) and anti-CTLA-4 (clone 9H10 BE0131) antibodies (9 mg/kg) or with the corresponding isotype sera (BioXCell, Lebanon, NH, USA) 3×/week starting on day 10 post inoculation (20).

### Lymph Node Collection and Immune Cell Isolation for Co-Cultures

Inguinal lymph nodes (LNs) were excised, weighed, and processed on ice for cell isolation: LNs were placed in 70 µm nylon mesh cell strainers propped over collection tubes. Cells were released by applying gentle pressure with the flat end of a syringe plunger, passed through the mesh, and rinsed with 3 ml ice-cold PBS. The dispersed cells were counted based on trypan blue exclusion and typically yielded 10^6^ intact cells/mg LN tissue. Isolated cells were collected by 300 g/8 min centrifugation at 4°C, mixed with freshly collected Yumm1.7 or YUMM1.7-CM cells at a ratio of 1:32 (target:effector) and seeded in black-wall 96-well plates. Typically, 10^4^ YUMM1.7 or YUMM1.7-CM cells were mixed with 3.2 × 10^5^00 lymph node cells and seeded in triplicate in 50 µg/ml Poly-L-lysine coated wells for 18-h co-incubation.

### Immunofluorescent Staining of Cultured Cells and Tumoral Cryosections

YUMM1.7 and YUMM1.7-CM cells were cultured in black-wall 96-well plates. After treatments, cells were fixed with 50 µl 4% PFA (20 min at 25°C), permeabilized/blocked for 30 min in 50 µl 5% goat serum/0.1% Triton X-100 in PBS, and incubated 2 h with primary antibodies (listed below). Following three washes with PBS/0.1% Tween-20, respective secondary antibodies (1:1,500 Alexa-488/594, Life Technologies) were applied for 30 min. For immunofluorescent analyses of tumoral sections**, t**umors were excised, snap frozen, embedded in Tissue-Tek OCT (#4583, Sakura Finetek), and cryo-sectioned at 10 μm. Cryosections were permeabilized by heat in citrate buffer (Dako S1699) for 18 min, blocked in PBS with 10% goat serum/0.3M glycine 45 min, and incubated 2 h with primary antibodies in PBS/1.5% goat serum: rat anti-CD8a (1:100, #100702, Biolegend), rat Granzyme B (1:100, ##488898-82, Invitrogen), rabbit Ki-67 (1:200, D3B5, #12202S, Cell Signaling), or rabbit Pfkfb3 (1:300, ab135820, Abcam). After washes with PBS/0.3% Tween-20, sections were incubated 45 min with anti-rabbit or anti-rat IgG dye conjugated antibodies Alexa-488/594 (Life Technologies), mounted with anti-fade reagent with DAPI, and observed with 10 or 40× objective using Olympus IX71 microscope equipped with QIC-F-M-12-C cooled digital camera (QImaging, Surrey, BC) with QCapture Pro (QImaging) software.

### EdU Incorporation and Detection

Tumoral cell proliferation was visualized by incorporation of the thymidine analog, 5-ethynyl-2’-deoxyuridine (EdU) (#146186, Abcam) into newly synthesized DNA. EdU was prepared in 0.9% NaCl and given IP at 50 mg/kg, 24 h prior to euthanasia. Tumors were excised and cryosections prepared as described above. For EdU fluorescence detection, the Click-IT™ EdU Alexa Fluor™-azide 594 Imaging Kit (Invitrogen, #C10339) was used per manufacturer’s protocol (21) and as we described (22). For double fluorescence detection, immunostaining was done ahead of applying the EdU detection solution (1:2,000) for 30 min/25°C in the dark. The number of positive cells/mm^2^ was quantified for four randomly selected fields in each section using ImageJ and mean value was calculated from four non-consecutive sections per mouse (n = 3); data are reported as mean ± SEM. Fluorescence was captured on Olympus IX71 fluorescent with QIC-F-M-12-C cooled digital camera. For *in vitro* studies, prior to co-culturing, YUMM1.7 and YUMM1.7-CM cells were pre-incubated with 10 µM EdU for 24 h to allow for EdU incorporation. After incubation cells were harvested, washed, mixed with LNs cells at 1:32 ratio, and plated in 96-well black wall plates for 18-h co-incubation. To visualize EdU positive cells, the Click-IT™ EdU Alexa Fluor™-azide 594 detection Kit (Invitrogen, #C10339) was used (as described above). Following three washes, in-well EdU fluorescence was read using the Tecan FL200 plate reader with Magellan™ software (Tecan, San Jose, CA, USA). Bright field/fluorescence images of co-cultures were captured and EdU positive nuclei were counted as we previously described (22). EdU positive nuclei were counted in randomly selected four fields per well for three co-incubation experiments; data are reported as mean ± SEM.

### TUNEL Assay

Tumoral cell death was detected *in situ* using the terminal deoxynucleotidyl transferase dUTP nick end labeling (TUNEL) kit (#11684795910, Roche). Briefly, cryopreserved tissue sections were permeabilized with 0.1% TritonX-100 in 0.1% sodium citrate solution for 8 min, washed with PBS, and incubated with reaction solution in humidified chamber for 1 h in the dark and mounted with Prolong Diamond anti-fade with DAPI; images were captured with Olympus IX71 fluorescent microscope.

### Real-Time (RT) qPCR Determination of mRNA Levels

The RNeasy plus mini kit (#74 134 Qiagen, Valencia, CA, USA) was used. Reverse transcription was with iScript RT supermix (#1708840 Bio-Rad) and RT-qPCR analyses with the CFX96 Real-Time System (Bio-Rad, USA). 18s mRNA was used as reference and relative expression levels were calculated using the formula: Relative expression = 2 ^[-(CT gene of interest − CT internal control)]^ according to Schmittigen & Litvak (23). PCR reactions were done in triplicate with SSO FAST EvaGreen^®^ supermix (#1725201 Bio-Rad). Cycling program was 95°C for 2 min, 40 cycles of two-step incubation, initially at 95°C 5 s then 15 s at 55°C followed by melting curve analysis. Primers are listed in **Supplemental Table S1**.

### Statistical Analysis

Data are provided as mean ± SEM calculated from three to four independent biological experiments, as stated. Unpaired two-tailed Student’s t-test was used to compare the means between groups. *P* value <0.05 was considered statistically significant. MegaStat^®^ software for Excel was used.

## Results

### Features of YUMM1.7-CM Clone Generated by *In Vitro* Exposure to Cisplatin

**Figure 1:**
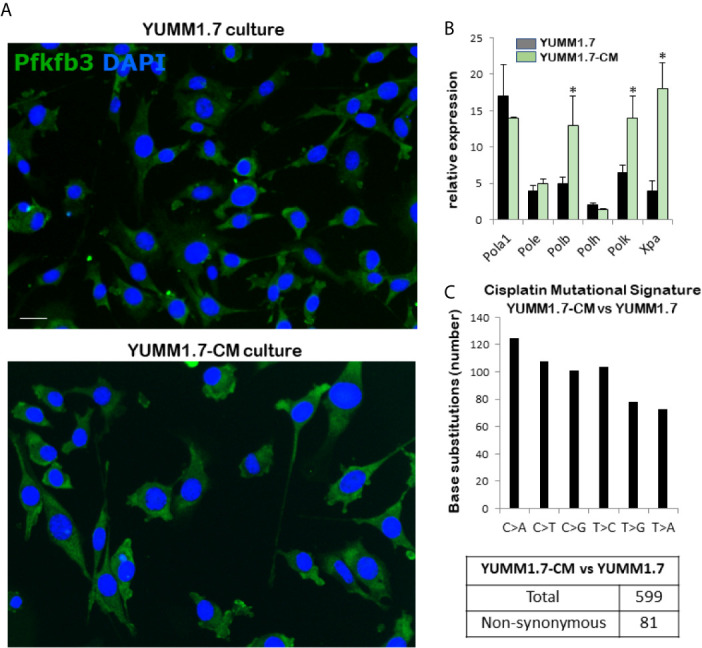
Properties of the cisplatin-mutagenized YUMM1.7-CM clone. **(A)** Representative micrographs show increased cellular dimensions of YUMM1.7-CM compared to parental YUMM1.7 cells. Cells are observed by immunofluorescence of the cytosolic glycolytic enzyme Pfkfb3 (green); nuclei stain blue with DAPI; scale bar, 10 µm. **(B)** Comparison of baseline expression of genes involved in DNA replication and repair. RNA isolated from three independent cultures of YUMM1.7 and YUMM1.7-CM cells was used to obtain mean ± SEM values; unpaired Student’s two-tailed t-test; *p < 0.05. **(C)** Cisplatin mutational signature: graphical representation of cisplatin-induced point mutations categorized by the type of base substitutions and synonymous and non-synonymous mutations.

Phenotypic changes observed in cultured cisplatin-mutagenized YUMM1.7-CM cells included increased cell size and reduced doubling time (**Figure 1A** and **Table 1**). The changes were visualized by nuclear DAPI stain and cytosolic immunofluorescence of the ubiquitous glycolytic enzyme, 6-phosphofructo-2-kinase/fructose-2,6-biphosphatase 3 (Pfkfb3) (**Figure 1A**). An approximately 25% reduction in cell doubling time was detected (**Table 1**). Interestingly, higher baseline expression of DNA repair proteins, including the base excision repair DNA polymerase beta, which is involved in oxidative DNA damage repair (24), the DNA damage recognition protein Xpa (25, 26), as well as the transcriptionally regulated error-prone translesion synthesis (TLS) DNA polymerase kappa (27), was observed (**Figure 1B**). The mutational signature of *in vitro* cisplatin exposure was determined from whole exome sequencing of parental YUMM1.7 melanoma cells and the mutagenized YUMM1.7-CM clone (**Figure 1C**). Comparison of the YUMM1.7-CM clone to parental cells revealed 599 cisplatin induced base substitutions (**Supplemental Table S2**), including 81 non-synonymous exonic mutations (**Supplemental Table S3**). Mutational signatures of cisplatin have been widely documented in different cancer cell lines and human tumors post cisplatin chemotherapy (28, 29).

**Table 1:** Distinctive features of mutagenized YUMM1.7-CM cells.

	YUMM1.7	YUMM1.7-CM	
**Nuclear metrics**	**mean ± SD (median)**	**mean ± SD (median)**	***P* value**
Radius (µm)	8.03 ± 1.9 (7.8)	9.92 ± 1.5* (9.8)	0.0015
Perimeter (µm)	50.1 ± 11.8 (49)	62.3 ± 9.3* (61)	0.0015
Surface Area (µm^2^)	212 ± 44 (189)	316 ± 58* (298)	0.0038
**Doubling time** (h)	18.2 ± 1.4	22.6 ± 1.6*	0.035

Measurements were obtained from four independent YUMM1.7 or Yumm1.7-CM cultures; *different from YUMM1.7; Student’s two-tailed unpaired t-test. Radius measurement = (short radius + long radius)/2.

### Augmented *Ex-Vivo* Cytotoxicity of Immune Cells Isolated From the YUMM1.7-CM Tumor-Draining Lymph Nodes (TDLNs)

**Figure 2:**
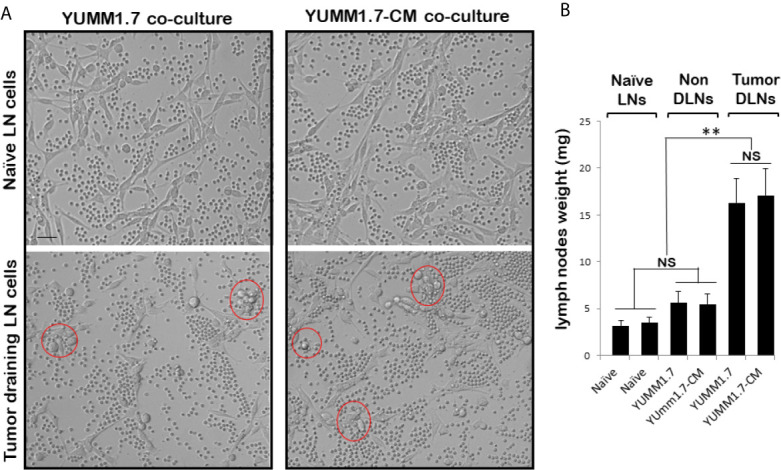
Ex-vivo cytotoxicity of immune cells isolated from lymph nodes (LNs). **(A)** Representative bright field images of YUMM1.7 and YUMM1.7-CM cells co-cultured with immune cells isolated from naïve non-tumor bearing mice (top) or from TDLNs of tumor-bearing mice (bottom). Examples of areas with immune cell clustering around tumor cells are delineated (red). Scale bar, 20 µm. **(B)** Graphical representation of mean weight of LNs excised from naïve and tumor-bearing mice (n = 6–10). Data are means ± SEM; unpaired Student’s two-tailed t-test; **p < 0.01; NS, not significant.

**Figure 3:**
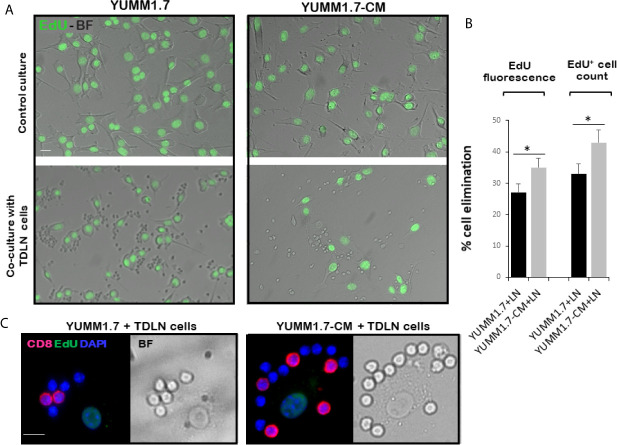
Enhanced ex-vivo cytotoxicity of cells isolated from TDLNs of YUMM1.7-CM compared to YUMM1.7 tumor-bearing mice. **(A)** Representative bright field (BF)/fluorescence micrographs of EdU-prelabeled YUMM1.7 and YUMM1.7-CM cells cultured alone (top) or co-cultured with immune cells isolated from respective TDLNs (bottom). EdU positive nuclei are observed in green. **(B)** Quantitation of YUMM1.7 and YUMM1.7-CM cells elimination following co-incubation with TDLNs cells measured by in-well EdU fluorescence readings or by EdU positive cell counts, as indicated. Data are presented as mean ± SEM obtained from four independent YUMM1.7-CM or YUMM1.7 cultures. Unpaired Student’s two-tailed t-test; *indicates different from YUMM1.7 co-cultures *p* < 0.05. **(C)** Representative micrographs of immune cells surrounding a tumor cell visualized by merged CD8 immunofluorescence (red)/BF images. EdU positive nuclei of YUMM1.7 or YUMM1.7-CM cells are observed in green; nuclei of TDLNs derived effector cells stain blue with DAPI. Scale bars, 10 µm.

**Figure 4:**
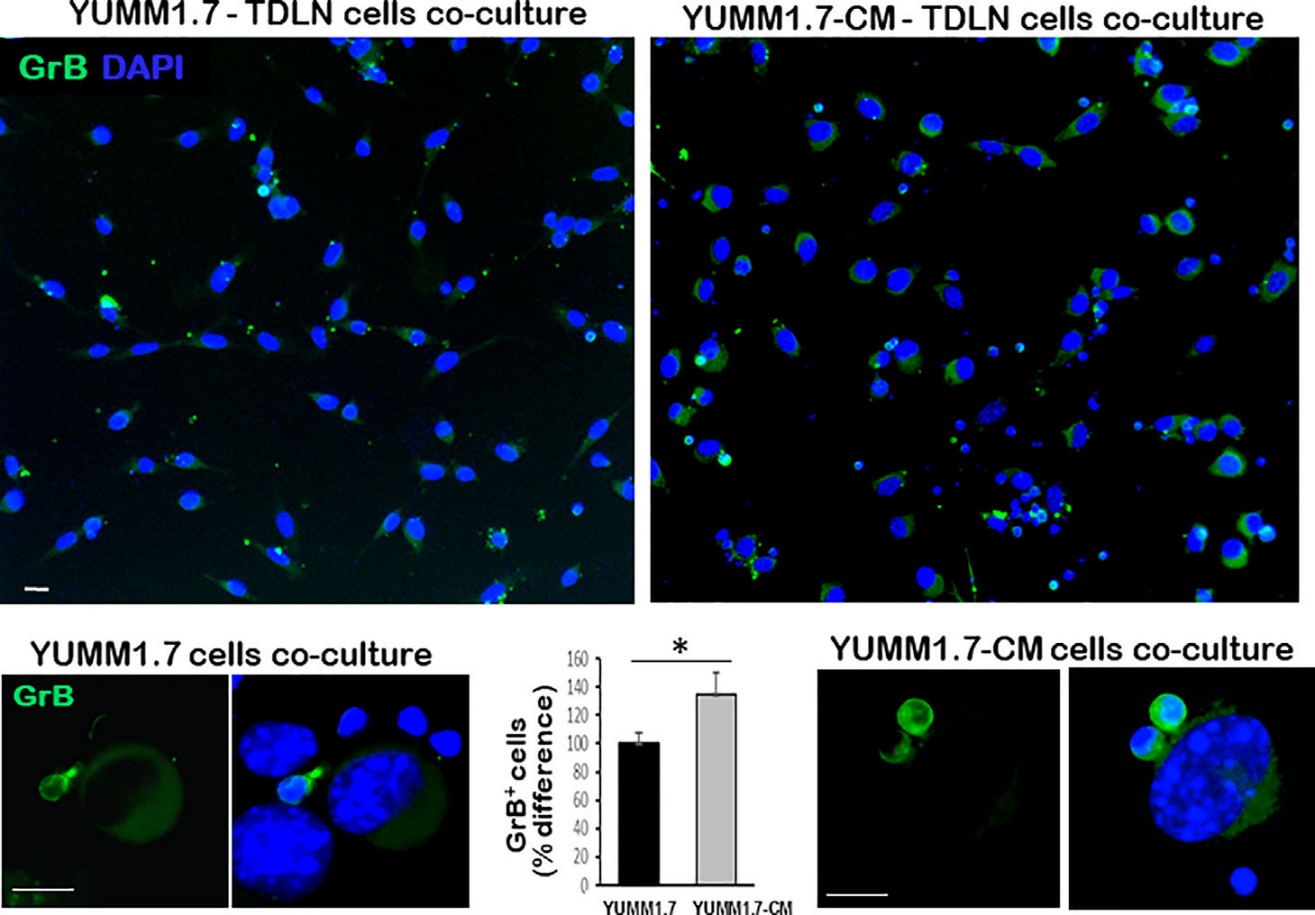
Higher Granzyme B (GrB) expression in co-cultures of TDLN cells with YUMM1.7-CM compared to YUMM1.7 cells. Representative micrographs of co-cultured YUMM1.7-CM and YUMM1.7 cells; GrB expression is visualized by immunofluorescence (green); nuclei stain blue with DAPI; scale bar, 10 µm. The difference in the number of GrB positive cells between YUMM1.7-CM and YUMM1.7 TDLNs was calculated from means ± SEM from four independent co-cultures; unpaired Student’s two-tailed t-test; *p < 0.05. Bottom: High magnification images of GrB expressing immune cells co-cultured with YUMM1.7 (left) and YUMM1.7-CM (right) tumor cells.

In co-cultures, clustering of immune cells isolated from the tumor draining lymph nodes, was observed around YUMM1.7 or YUMM1.7-CM cells. In contrast, clustering was not seen with immune cells isolated from lymph nodes of naïve non-tumor bearing mice. Bright field images of co-cultures seeded at a 1:32 ratio of target:effector cells show clustering of TDLN-derived immune cells around cultured YUMM1.7 and YUMM1.7-CM cells (**Figure 2A**, examples demarcated in red). The immune system engagement was also reflected in three-fold weight increases of TDLNs compared to non-DLNs or to LNs collected from naïve non-tumor bearing mice (**Figure 2B**). This is consistent with TDLN enlargement reported in patients (30) and recapitulated in mouse models (31, 32). Interestingly, dependence of anti-PD-1/PD-L1 therapy success on extant tumor draining lymph nodes, was recently reported, suggestive of clinical benefits while performing ICB prior to TDLN resection (33, 34). Here, we observed that immune cells isolated from the YUMM1.7-CM tumor-bearing mice TDLNs, were more effective at eliminating co-cultured target cells compared to cells from TDLNs of YUMM1.7 tumor-bearing mice. For quantitative assessments, prior to co-culturing, tumor cells were pre-incubated in the presence of the thymidine analog, EdU (10 µM) to allow for EdU incorporation into nuclear DNA and subsequent florescent detection of tumor cells. After 24-h incubation, cells were harvested and re-plated in EdU-free growth medium, either alone (control), or after mixing with TDLN derived cells for 18-h co-incubation (**Figure 3A**). Next, the extent of EdU-positive cells elimination from co-cultures was assessed using two methods: 1) measurement of in-well EdU fluorescence and 2) counting EdU positive cells (**Figure 3B**). While cell counting was slightly more sensitive than in-well fluorescence readings, results obtained with both methods, revealed greater elimination of YUMM1.7-CM compared to YUMM1.7 cells following co-incubation with the respective TDLNs-derived immune cells (44 ± 4% for YUMM1.7-CM cells *versus* 35 ± 3% for YUMM1.7 cells). These findings agree with immunofluorescent imaging that revealed greater presence of CD8^+^ T cells among lymphocytes surrounding the YUMM1.7-CM cells (**Figure 3C**). Higher levels of granzyme B positivity in co-cultures with cells isolated from TDLNs of YUMM1.7-CM tumors-bearing mice were also observed (**Figure 4**). Granzyme B protease is a key mediator of target cell elimination by cytotoxic CD8 T lymphocytes (35).

### Curtailed Tumoral Cell Proliferation in YUMM1.7-CM- Compared to YUMM1.7-Generated Tumors

**Figure 5:**
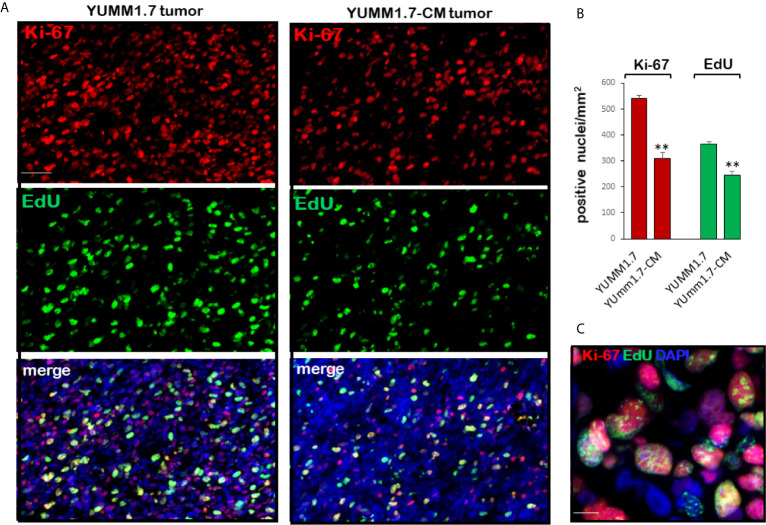
Proliferating tumoral cells are detected by Ki-67 immunofluorescence and EdU incorporation into nascent tumoral DNA. **(A)** Representative micrographs of tumoral cryosections show Ki-67 positive nuclei (top, red) and EdU incorporating-nuclei (center, green); bottom, double stained images merged with DAPI (blue). Ki-67 and EdU positivity is greater in YUMM1.7 than in YUMM1.7-CM tumors. **(B)** Bar graphs represent Ki-67 and EdU positive nuclei/mm2 in four non-consecutive cryosections/tumor analyzed for three mice/group presented as mean ± SEM, unpaired Student’s two-tailed t-test; **p < 0.01; scale bar, 50 µm. **(C)** Merged Ki-67/EdU/DAPI high magnification image; scale bar, 10 µm.

*In vivo* proliferation of tumoral cells was assessed by immunodetection of the proliferation biomarker Ki-67, which is expressed in cycling cells and identifies the proliferating fraction in a given cell population (36), and by detection of tumor cells that incorporate EdU into nascent DNA. Analyses of tumoral cryosections revealed higher numbers of Ki-67 positive cells in the YUMM1.7 when compared to YUMM1.7-CM generated tumors (**Figures 5A, B**). Similarly, the number of EdU incorporating tumoral cells was higher in YUMM1.7 compared to YUMM1.7-CM tumors (**Figures 5A, B**). The complex nuclear patterns of Ki-67 expression and EdU incorporation were observed at high magnification in merged images (**Figure 5C**), reflecting heterogeneity of tumoral cells transitioning through the different stages of the cell cycle.

### Higher Levels of Tumor Infiltrating CD8 T Lymphocytes and Granzyme B Expression in YUMM1.7-CM Compared to YUMM1.7-Generated Tumors

**Figure 6:**
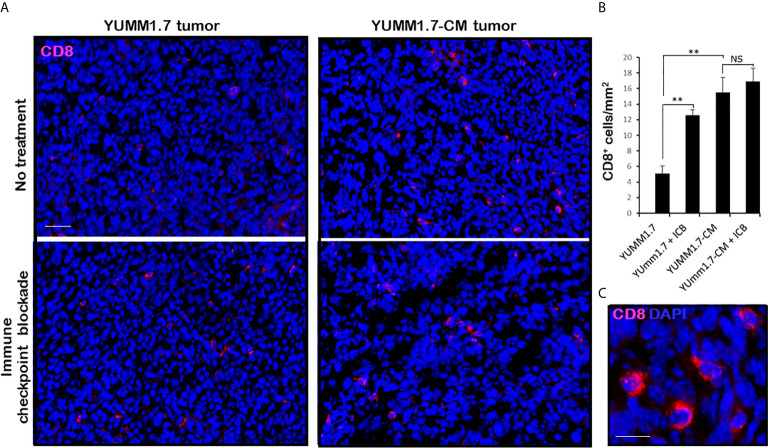
Markedly higher levels of tumor infiltrating CD8 T lymphocytes in YUMM1.7-CM compared to YUMM1.7 generated tumors. **(A)** Red immunofluorescence identifies CD8+ T cells in tumor cryosections; nuclei stain blue with DAPI; scale bar, 50 µm. **(B)** Quantification of infiltrating CD8+ T cells in the indicated groups. Bars represent mean ± SEM number of CD8 cells derived from four non-consecutive sections/tumor analyzed for three mice/group. Unpaired Student’s two-tailed t-test; **p < 0.01; NS, not significant. **(C)** High magnification; scale bar, 10 µm.

**Figure 7:**
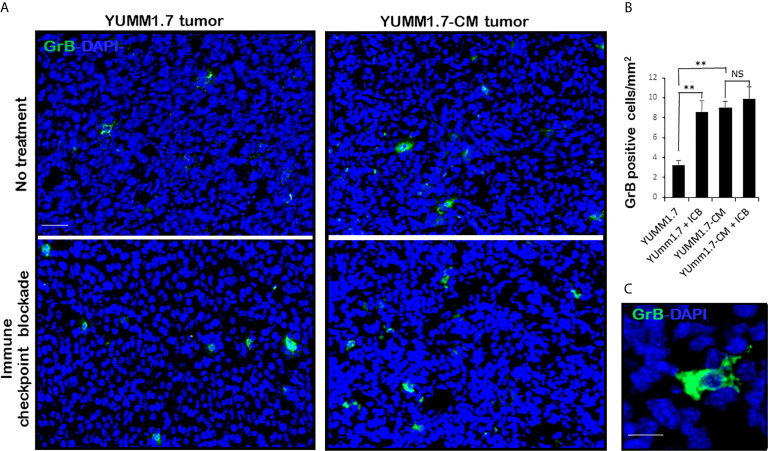
Greater expression of Granzyme B in YUMM1.7-CM compared to YUMM1.7 generated tumors. **(A)** Representative micrographs show GrB immunofluorescence (green) in tumoral cryosections. More GrB positive cells with intensified diffuse staining patterns are detected in YUMM1.7-CM when compared to YUMM1.7 tumors and in YUMM1.7 tumors following ICB regimen compared to untreated YUMM1.7 tumors. **(B)** Fields from four nonconsecutive sections/tumor (n = 3) per group were evaluated; scale bar, 50 µm. Data are presented as mean ± SEM and compared using unpaired Student’s two-tailed t test, **p < 0.01, NS, not significant. **(C)** High magnification shows diffuse pattern of GrB immunofluorescence; scale bar, 10 µm.

Mice inoculated with either parental YUMM1.7 or mutagenized YUMM1.7-CM melanoma cells according to our standard protocol (13), were randomly assigned to receive IP, isogenic or anti-CTLA-4/anti-PD-1 sera 3×/week (n = 6–10). Tumors and lymph nodes were collected 20 days post inoculation. Analyses of tumoral cryosections revealed ~3-fold higher baseline levels of infiltrating CD8^+^ T lymphocyte (**Figure 6**) and granzyme B expression (**Figure 7**) in YUMM1.7-CM when compared to YUMM1.7-generated tumors. Interestingly, ICB treatment of mice bearing the YUMM1.7 tumors, resulted in marked increases in tumor infiltrating CD8 lymphocytes and granzyme B expression, nearing the baseline observed in YUMM1.7-CM generated tumors (**Figures 6** and **7**, bottom panels). In contrast, when ICB was administered to the mutagenized YUMM1.7-CM tumors-bearing mice, the treatment resulted in only marginal changes in the number of infiltrating CD8 T lymphocytes (**Figure 6**) and granzyme B expression levels (**Figure 7**).

### YUMM1. 7-CM and YUMM1.7-Generated Tumors Differ in Growth Profiles, the Extent of Tumoral Cell Death, and Responses to ICB Regimen

**Figure 8:**
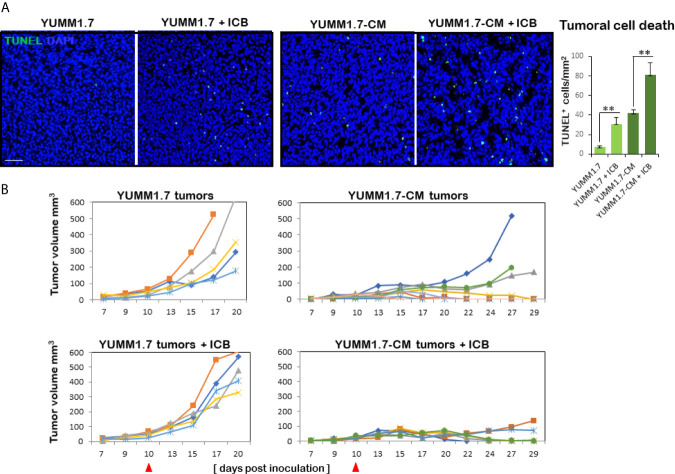
Higher rate of cell death and growth suppression in YUMM1.7-CM compared to YUMM1.7-generated tumors. **(A)** Tumoral cell death detected by TUNEL assay (green); nuclei stain blue with DAPI; scale bar, 50 µm. Right, graphic representation of TUNEL positivity levels. Values are derived from four non-consecutive sections/tumor for three mice/group and presented as mean ± SEM; unpaired Student’s two-tailed t-test; **p < 0.01. **(B)** Individual growth curves of tumors in the indicated groups (n = 5–6). Left, in YUMM1.7-generated tumors growth rates are not restricted by ICB (bottom). Right, YUMM1.7-CM generated tumors have slower growth rates with occasional spontaneous regression. YUMM1.7-CM tumors growth is suppressed by ICB regimen (bottom). Arrowheads mark the start day of ICB regimen.

The extent of tumoral cell death assessed by TUNEL assay, revealed significantly higher baseline levels of TUNEL positive cells in YUMM1.7-CM compared to YUMM1.7-generated tumors, as well as marked increases in TUNEL positivity following ICB regimen (**Figure 8A**). Individual tumor growth curves for the different groups revealed slower growth rates of YUMM1.7-CM compared to YUMM1.7 tumors (**Figure 8B**). In the fast-growing YUMM1.7 cohort, all tumors were collected for analyses 20 days post inoculation when tumor volumes reached about 500 mm^3^. For comparative analyses, half of the YUMM1.7-CM cohort, was similarly processed at 20 days post inoculation for tumor and lymph node collection, while for the remining mice, tumor growth rates continued to be monitored (n = 6). Importantly, in the YUMM1.7-CM tumor-bearing mice/ICB treatment group, nearly all tumors regressed, while growth of YUMM1.7 tumors was not suppressed by the ICB regimen (**Figure 8B**). This is consistent with our earlier findings, that demonstrated that a combination protocol that includes ICB in conjunction with intratumoral cisplatin delivery is required for eradication of YUMM1.7 melanoma tumors (13).

## Discussion

**Figure 9:**
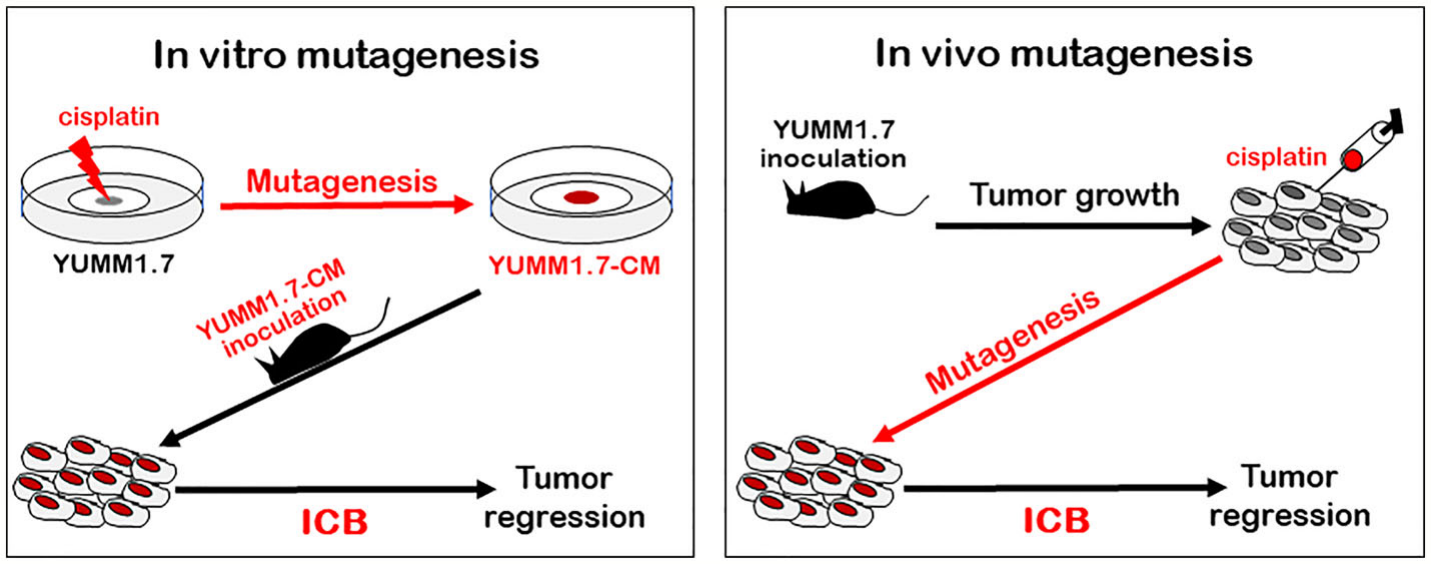
Outline of two different mutagenesis-based protocols that exert ICB-mediated tumor regression in mouse model of melanoma: Tumors generated with in *vitro* mutagenized YUMM1.7-CM melanoma cells (left, red), as well as parental YUMM1.7-generated tumors, which are intratumorally injected with cisplatin (right, red) regress in response to ICB treatment (ICB regimen alone fails to suppress growth of YUMM1.7 tumors).

Our study demonstrates that tumors generated with *in vitro* mutagenized YUMM1.7-CM cells elicit a productive antitumor immune response *in vivo*, in the mouse (**Figure 9**). Consistent with enhanced immune surveillance in immunocompetent mice, growth rates of the cisplatin-mutagenized YUMM1.7-CM-generated tumors are reduced. Furthermore, immune cells isolated from YUMM1.7-CM tumors draining lymph nodes are more potent *ex-vivo* at eliminating co-cultured tumor cells, when compared to cells isolated from lymph nodes draining the parental YUMM1.7 tumors. Importantly, we show that the YUMM1.7-CM tumors readily regress in response to immune checkpoint blockade (ICB) regimen, similarly to complete response to ICB achieved in YUMM1.7 tumor bearing mice when ICB is administered in conjunction with low-dose intratumoral cisplatin delivery, as we previously reported (13). In the current study, we characterized the mutational signature of cisplatin and described phenotypic changes detected in cultured YUMM1.7-CM cells, and in the YUMM1.7-CM-generated tumors. Whole exome sequencing of the mutagenized YUMM1.7-CM clone revealed that nearly 15% of the acquired base substitutions, cause amino acid changes that are likely to contribute to neoantigen formation and improved engagement of immune system.

The notion that neoantigen formation enhances antitumor immunogenicity and improves responses to ICB therapy is supported by clinical outcomes demonstrating that tumors with high mutational loads are more likely to respond to ICB therapies (2, 37), rendering the mutational burden an important, albeit imperfect predictor of response to ICB (3, 4, 38, 39). Examples from the clinic include subpopulations of colon cancer patients with DNA mismatch repair deficiency characterized by high tumoral mutation burden and elevated neoantigens, which have shown high response rates to anti-PD-1 checkpoint blockade (7, 40, 41). Similar observations were made in melanomas (8, 42, 43) and lung cancers (44, 45). These encouraging clinical outcomes were recapitulated in animal studies that sought to investigate the links between mutational loads and efficacy of responses to ICB. One approach included the use of CRISPR-Cas9 to inactivate different components of the DNA mismatch repair pathway in several mouse cancer models, which led to significant increases in mutational loads and neoantigen formation in the modified cells, resulting in augmented immune surveillance and restricted growth of subsequently formed tumors (46). Other experimental approaches involved the use of a cyclin-dependent kinase 7 inhibitor to induce replication stress and genomic instability, which resulted in enhanced response to ICB in mouse lung cancer (47), and in mouse melanoma model, the use of UVB-mutagenized YUMM1.7 cells to generate tumors, resulted in favorable response to ICB treatment (20).

We previously developed a mouse melanoma combination treatment protocol that entails ICB regimen given in conjunction with low-dose intratumoral cisplatin delivery designed to block high fidelity replicative DNA synthesis in tumoral cells, while activating the mutagenic DNA damage tolerance pathway (13). The DNA damage tolerance pathway involves a shift to TLS DNA polymerases-catalyzed mutagenic synthesis, which is critical for averting the replication forks collapse following formation of DNA synthesis-blocking cisplatin:DNA crosslinks (6, 11, 48). The error prone TLS polymerases eta (49–51) and kappa (52, 53) have been specifically implicated in bypass synthesis of cisplatin crosslinks (54–57), and we detected upregulation of these polymerases in melanoma tumors following intratumoral cisplatin delivery (13). Consistent with the premise that mutagenic DNA synthesis increases mutational loads (58, 59) and neoantigen formation, thereby augmenting tumor immunogenicity and antitumor surveillance (7, 60), tumor eradication was achieved with the intratumoral cisplatin delivery/ICB combination protocol that we developed (13).

Here we demonstrate that durable tumor regression is achieved with the *in vitro* mutagenized YUMM1.7-CM cell-generated tumors in mice subjected to the ICB regimen (**Figure 9**, left). Involvement of TDLNs in the antitumor immune response is manifested *ex-vivo*, as augmented potency of the YUMM1.7-CM TDLN derived immune cells at eliminating the co-cultured tumor cells. The corresponding *in vivo* experiments reveal a markedly greater infiltration of CD8 T lymphocytes and higher granzyme B expression in the TME of YUMM1.7-CM tumors compared to TME of YUMM1.7-generated tumors. Interestingly, while ICB regimen leads to marked increases in the levels of infiltrating CD8 T lymphocytes and granzyme B expression in the YUMM1.7-generated tumors, similar increases are not observed in YUMM1.7-CM tumors. Although speculative, it is plausible that as previously suggested (33, 61), the ICB treatment may not only reinvigorates extant immune cells in the TME but also potentiates trafficking of T lymphocytes to the TME, which might be the case with the less immunogenic YUMM1.7 tumors. Conversely, in the YUMM1.7-CM tumors, ICB might function chiefly to reinvigorate cytotoxic T lymphocytes, which had been already recruited to the TME due to augmented immunogenicity of the YUMM1.7-CM tumors, facilitating regression of YUMM1.7-CM tumors in response to ICB regimen. Collectively, our findings support the premise that buildup of tumoral mutations driven by an activation of intrinsic tumoral DNA damage tolerance pathway enhances immunogenicity and immune surveillance of solid tumors, charting a path to improve success rates of chemotherapy/ICB combination protocols in the clinic. Since the proposed new protocol relies on low-dose intratumoral delivery of a common chemotherapeutic drug combined with checkpoint blockade, which is currently one of the most successful modes of anticancer therapy, potential translatability of this combination protocol to the clinic warrants thorough investigation.

## Data Availability Statement

The raw sequencing data from this study have been submitted to NCBI Sequence Read Archives (SRA) under accession number PRJNA734588.

## Ethics Statement

The animal study was reviewed and approved by the Animal Care and Use Committee of the University of Texas Medical Branch.

## Author Contributions

EE and DT conceptualized and developed the study. EE developed the experimental design. FM performed the experiments and data analyses. SW designed and conducted the sequencing data analyses and interpretation. EE analyzed and interpreted the data and wrote the manuscript. All authors contributed to the article and approved the submitted version.

## Conflict of Interest

The authors declare that the research was conducted in the absence of any commercial or financial relationships that could be construed as a potential conflict of interest.

## Supplementary Material

Supplementary Table 2 Cisplatin mutations identified by the GATK pipeline and Mutect2. Mutations were identified and filtered as described in the methods. All mutations, including those in the exon flanking regions that passed the Mutect2 filter are included. Chromosomes and base numbering are based on the mouse mm10 reference genome.

Supplementary Table 3 Effects of cisplatin induced exonic mutations. Mutations were identified as described in the methods. The chromosome (chr), position (base), and gene for each change is indicated. The effect of the change at the codon level is shown in the codon change column and may be a codon change, insertion, deletion, or a change at the splice site. Two mutations were identified in the 3’ untranslated part of the last exon. The effect at the protein level is indicated in the effect column. A synonymous codon change is indicated by nc (no change), and changes creating a stop codon are indicated by amber or ochre.
