# Supplementary material for: Enhanced Antitumor Response to Immune Checkpoint Blockade Exerted by Cisplatin-Induced Mutagenesis in a Murine Melanoma Model
Source: Front Oncol. 2021 Jul 6;11:701968. doi: 10.3389/fonc.2021.701968 (PMC8290318; doi:10.3389/fonc.2021.701968)
Supplement: Supplementary file 1 [file Table_1.docx]

**Suppl Table 1**. IDs and sequences of mouse primers.

| **Gene Symbol** | **Forward** | **Reverse** |  | **Accession number** | **Gene name** |
| --- | --- | --- | --- | --- | --- |
| 18s | gtaacccgttgaaccccatt | ccatccaatcggtagtagcg |  | **NR_003278.3** | **Mus musculus 18S ribosomal RNA** |
| Pola1 | gccatcgacacccaatactac | tctaaattgagtggagtcaagtcct |  | **NM_008892.2** | **Polymerase alpha 1 (Pola1)** |
| Polb | ctcgagttactggcattggac | ccttcatctacaaacttccttgc |  | **NM_011130.2** | **Polymerase (DNA directed), beta (Polb)** |
| Pole | tcaagactttgatattcggacaat | cactccccagcctctctatg |  | **NM_011132.2** | **Polymerase epsilon (Pole)** |
| Polh | agggaaaacagctctggcta | tccagagctaactgcaaaagc |  | **NM_030715.3** | **Polymerase eta (Polh)** |
| Polk | cccaaagaaagctcgagaag | ttcttcaaagttgagctctttgtct |  | **NM_012048.2** | **Polymerase kappa (Polk)** |
| Xpa | acgagattggaaacattgttca | ctctttcccgcattcttcac |  | **NM_011728.2** | **Xeroderma pigmentosum, complementation group A (Xpa)** |

|  |  |  |  |  |  |  | |
| --- | --- | --- | --- | --- | --- | --- | --- |
|  |  |  |  |  |  |  |  |
